# Long-Term Cognitive Impairment After CAR-T Therapy Versus Autologous Stem Cell Transplantation: A Propensity Score-Matched Cohort Study

**DOI:** 10.3390/diagnostics16121862

**Published:** 2026-06-16

**Authors:** Anna Blyzniuk, Po-Huang Chen, Wei-Cheng Chang, Hsin-Yu Chen, Li-Ting Kao, Tina Yi-Jin Hsieh, Ming-Shen Dai, Hong-Jie Jhou, Cho-Hao Lee

**Affiliations:** 1 School of Medicine, O.O. Bogomolets National Medical University, 01601 Kyiv, Ukraine; annablyznjuk@gmail.com; 2 Division of Hematology and Oncology, Department of Internal Medicine, Tri-Service General Hospital, National Defense Medical University, Taipei 11490, Taiwan; chenpohuang@hotmail.com (P.-H.C.); dms1201@gmail.com (M.-S.D.); 3 Department of Oncology, Tri-Service General Hospital, National Defense Medical University, Taipei 11490, Taiwan; 4 Department of Ophthalmology, Universe Eye Center, Hsinchu 30055, Taiwan; cwc761229@gmail.com; 5 Department of Family Medicine, Chi Mei Medical Center, Tainan 71004, Taiwan; yupucca@gmail.com; 6 Graduate Institute of Life Sciences, National Defense Medical University, Taipei 11490, Taiwan; kaoliting@mail.ndmutsgh.edu.tw; 7 School of Pharmacy, National Defense Medical University, Taipei 11490, Taiwan; 8 Department of Pharmacy Practice, Tri-Service General Hospital, Taipei 11490, Taiwan; 9 Department of Obstetrics & Gynecology, Beth Israel Deaconess Medical Center, Boston, MA 02215, USA; hsiehyijin@gmail.com; 10 Neurological Institute, Changhua Christian Hospital, Changhua 50006, Taiwan

**Keywords:** CAR-T cell therapy, autologous stem cell transplantation, cognitive impairment, immune effector cell-associated neurotoxicity syndrome (ICANS), neurotoxicity, propensity score matching, real-world evidence, TriNetX, hematological malignancies

## Abstract

**Background/Objectives**: Chimeric antigen receptor T-cell (CAR-T) therapy has transformed outcomes in relapsed or refractory hematologic malignancies, but long-term cognitive outcomes remain poorly understood. We compared the incidence and time course of cognitive impairment and associated neurological complications after CAR-T therapy compared with autologous stem cell transplantation (ASCT). **Methods**: This retrospective, propensity-matched cohort study utilized the TriNetX US Collaborative Network (January 2014–April 2025). To ensure concurrent comparisons, ASCT recipients were restricted to an index date beginning in August 2017 or later. CAR-T recipients were matched 1:1 to ASCT recipients for demographics, disease, comorbidities, prior and concomitant treatments, and laboratory parameters. The primary endpoint was time to cognitive impairment, as defined by ICD-10 codes. **Results**: After comparing 3067 CAR-T patients (median follow-up 634 days) with 3067 ASCT patients (median follow-up 713 days), CAR-T recipients had a higher risk of cognitive impairment (HR 1.58; 95% CI 1.39–1.80; *p* < 0.001). Because the risks were not proportional (Schaenfeld *p* < 0.001), the difference was also expressed as restricted median survival time (RMST): CAR-T recipients spent approximately 25 and 53 days fewer days without cognitive impairment at 1 and 2 years, respectively (both *p* < 0.001). The risk was greatest at 30 days (HR 4.22; 95% CI 3.23–5.53), but remained elevated in control analyses at 30 and 90 days that excluded the acute ICANS window (HR 1.30 and 1.25, respectively; both *p* < 0.05). Neurological dysfunction, particularly encephalopathy (HR 2.04; 95% CI 1.73–2.40), was more common after CAR-T. Conversely, CAR-T was associated with a reduced risk of secondary acute myeloid leukemia (HR 0.46; 95% CI 0.38–0.55; *p* < 0.001). **Conclusions**: CAR-T therapy is associated with a higher risk of cognitive impairment that persists beyond the acute phase. As these are observational, code-based data, they should be interpreted as associations rather than evidence of a specific mechanism, and they highlight the need for informed consent discussions, long-term neurocognitive monitoring, and the development of neuroprotective strategies.

## 1. Introduction

Chimeric antigen receptor T-cell (CAR-T) therapy has revolutionized the treatment of relapsed and refractory hematological malignancies, demonstrating significantly superior efficacy compared with traditional salvage regimens [1,2,3]. Randomized clinical trials, including ZUMA-7 and TRANSFORM, have confirmed the benefits of CAR-T in the treatment of diffuse large B-cell lymphoma, challenging the traditional role of autologous stem cell transplantation (ASCT) [4,5].

Despite its therapeutic success, CAR-T is associated with specific toxicity. Immune cell-associated neurotoxicity syndrome (ICANS) occurs in a significant proportion of patients and is usually reversible [6,7]. However, emerging evidence suggests that cognitive impairment may persist beyond the acute phase, as evidenced by patient surveys and experimental studies demonstrating long-term neuroinflammatory changes [8,9,10]. In comparison, ASCT has well-described long-term complications, including cognitive dysfunction, but systematic risk comparisons between CAR-T and ASCT are lacking [11,12].

To fill this gap, we conducted a large cohort study using propensity score matching to compare the incidence and time course of cognitive impairment and associated neurological complications in patients after CAR-T compared with ASCT. The results have practical implications for treatment selection, patient counseling, and long-term care planning for oncohematological patients.

## 2. Materials and Methods

### 2.1. Study Design and Data Source

We conducted a retrospective cohort study using the TriNetX US Collaborative Network, which contains anonymized electronic medical records from over 100 healthcare organizations [13]. The study period extended from January 2014 to April 2025. The study was reported in accordance with the STROBE statement and its RECORD extension for routinely collected health data [14,15]. Institutional ethics approval was not required, as only anonymized, HIPAA-compliant aggregate data were analyzed. The study protocol was retrospectively deposited on the Open Science Framework (OSF DOI: 10.17605/OSF.IO/2BXR7; https://osf.io/2bxr7; accessed 25 May 2026) as a transparency record of the prespecified cohort and outcome definitions, covariates, and analysis plan, and to tag explicitly the analyses added in response to peer review; the absence of prospective registration is discussed in Section Limitations.

### 2.2. Patient Identification

CAR-T Cohort. Patients were identified using drug names and Healthcare Common Procedure Coding System (HCPCS) codes for all FDA-approved CAR-T products: tisagenlecleucel (Kymriah), axicabtagene ciloleucel (Yescarta), lisocabtagene maraleucel (Breyanzi), brexucabtagene autoleucel (Tecartus), idecabtagene vicleucel (Abecma), and ciltacabtagene autoleucel (Carvykti). The index date was the date of CAR-T infusion. Complete codes are provided in Supplementary S2.

**Figure 1:**
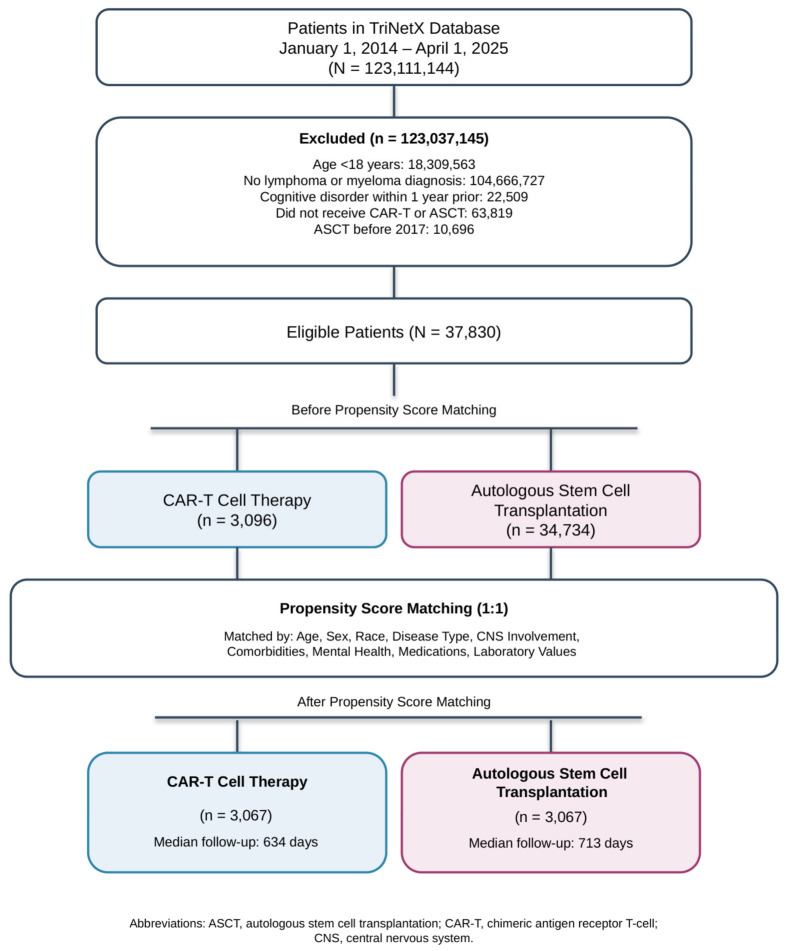
Study cohort selection. Flow diagram showing patient selection from the TriNetX database. Of 123,111,144 patients screened, 37,830 met eligibility criteria (including restriction of ASCT to an index date on or after August 2017) and, after propensity score matching, 3067 CAR-T patients were matched to 3067 ASCT patients.

ASCT Cohort. Patients were identified using CPT code 38241 and ICD-10-PCS code 30233G0. The index date was the date of stem cell infusion. To address calendar-era differences in supportive care, coding practice, and patient selection, ASCT recipients were restricted to an index date on or after August 2017 (the first FDA CAR-T approval), yielding a contemporaneous comparator (Figure 1) [16].

### 2.3. Eligibility Criteria

Inclusion criteria: age ≥ 18 years at index date; diagnosis of lymphoma or multiple myeloma; receipt of CAR-T therapy or ASCT during the study period; availability of records for at least 6 months before index; and at least 30 days of follow-up after index.

Exclusion criteria: previous cognitive impairment, dementia, or related conditions within 1 year before index; incomplete data; ASCT index date before August 2017; and receipt of both CAR-T and ASCT during the study period.

### 2.4. Outcomes

Primary Outcome. Composite cognitive impairment, defined as the first post-index occurrence of any of the following ICD-10 codes: R41.81, R41.89, R41.3, R41.840, R41.82, F03.90, and R45.81. These codes were pooled because individual cognitive codes are uncommon and because clinical cognitive impairment manifests heterogeneously across memory, attention, and executive domains; the composite increases statistical power while individual-component analyses confirm that the signal is not driven by any single code. A prespecified sensitivity definition removed R41.82 (altered mental status)—the least specific component—to test specificity for chronic impairment. Delirium and acute confusional states were excluded from the primary composite to maintain specificity for cognitive rather than encephalopathic outcomes.

Secondary Outcomes. Neurological dysfunction, individual neurological complications, mood and stress-related disorders, functional outcomes (falls, mobility), secondary malignancies (acute myeloid leukemia, myelodysplastic syndrome, secondary T-cell lymphoma), coagulation abnormality, venous thromboembolism, and negative control outcomes (gallstones, kidney stones, appendicitis). The negative controls were chosen because they are common enough in this oncology population to provide statistical power, have no plausible inflammatory, cytotoxic, or neuro-immune pathway shared with either CAR-T or ASCT, and are typically detected through acute symptomatic presentation rather than active surveillance, so that equal incidence between arms supports absence of differential surveillance, detection, or coding bias. Complete ICD-10 definitions are provided in Supplementary Table S1.

### 2.5. Propensity Score Matching

Patients were matched 1:1 using a nearest-neighbor algorithm without replacement (caliper 0.2 × SD of the logit of the propensity score). Covariates included demographics (age, sex, race), primary diagnosis, central nervous system involvement, comorbidities, mental health conditions, prior and concomitant disease-directed treatments (systemic corticosteroids, rituximab, bendamustine, daratumumab, lenalidomide, carfilzomib, bortezomib, polatuzumab vedotin, brentuximab vedotin, intrathecal chemotherapy, antineoplastic radiation therapy), and laboratory values (body mass index; lactate dehydrogenase [LDH]). LDH was modeled as a categorical variable (≤250, 251–500, ≥500 U/L) rather than continuous, because extreme values produced residual imbalance (continuous LDH SMD 0.141); categorization removed this imbalance. Balance was assessed by standardized mean differences (SMD < 0.1; Table 1). Full details are in Supplementary S4.

### 2.6. Statistical Analysis

Baseline characteristics were compared using chi-square and *t*-tests. Cumulative incidence was estimated as 1−Kaplan–Meier survival and compared with log-rank tests; Cox proportional-hazards models provided hazard ratios (HR) with 95% confidence intervals (CI). Cumulative incidence and the cumulative (index-to-landmark) hazard ratio were reported at fixed landmarks (Table 2a). The proportional-hazards assumption was evaluated with the Schoenfeld residual global test; because it was violated, the single Cox HR was retained only as a time-averaged summary, and the restricted mean survival time (RMST) difference in cognitive-impairment-free days at 1, 2, and 3 years was used as the principal, assumption-free effect measure (Table 2c) [17,18,19,20]. RMST and its variance were derived from the Kaplan–Meier estimates, with patient-level risk sets reconstructed by the method of Guyot et al. [20]. Patients were censored at death, loss to follow-up (>180 days without an encounter), or end of study (1 April 2025). To address delayed ICANS, landmark analyses re-anchored follow-up at Day 30 (primary) and Day 90 (Table 2b); analyses excluding events within 7, 14, and 21 days are reported in Supplementary Table S2 [16,21,22]. Subgroup analyses were stratified by age, sex, race, diagnosis, and CAR-T product. E-values were calculated to assess robustness to unmeasured confounding [23,24]. All tests were two-sided with α = 0.05.

## 3. Results

### 3.1. Baseline Characteristics

Of 37,830 patients meeting inclusion criteria in the TriNetX network, 3067 CAR-T therapy patients were successfully matched to 3067 ASCT patients (Figure 1). Groups were well-balanced in demographics, diagnoses, comorbidities, prior treatments, and baseline laboratory values (all SMD < 0.1; Table 1). The median age was 60 years, and 61% were male. The most common diagnoses were diffuse large B-cell lymphoma (57%) and multiple myeloma (33%). The median follow-up was 634 days for CAR-T and 713 days for ASCT, reflecting the more recent introduction of CAR-T.

### 3.2. Primary Outcome: Cognitive Impairment

**Figure 2:**
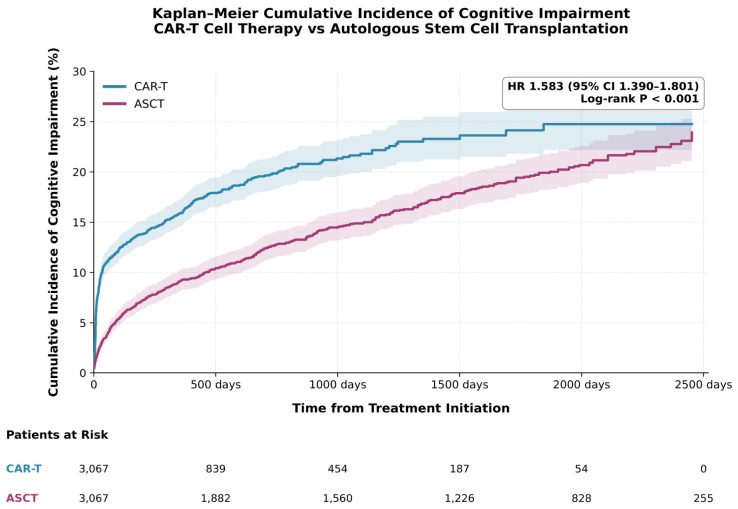
Kaplan–Meier cumulative incidence of cognitive impairment. Cumulative incidence curves comparing cognitive impairment between CAR-T cell therapy and ASCT recipients over 2500 days of follow-up. Shaded areas represent 95% confidence intervals. CAR-T recipients showed a consistently higher cumulative incidence throughout the follow-up period (HR 1.58; 95% CI 1.39–1.80; log-rank *p* < 0.001).

CAR-T patients had a higher cognitive impairment risk compared with ASCT recipients (HR 1.58; 95% CI 1.39–1.80; *p* < 0.001; E-value 2.54). The cumulative incidence curves showed an early and sustained difference between groups (Figure 2): at 180 days, the incidence was 13.7% versus 6.9% (absolute difference 6.8%), and at 1000 days 21.3% versus 14.5% (absolute difference 6.8%) (Table 2a). Because the proportional-hazards assumption was violated (Schoenfeld *p* < 0.001), the difference was summarized using RMST: CAR-T recipients remained free of cognitive impairment for 24.8 fewer days at 1 year (95% CI −30.1 to −19.6) and 53.4 fewer days at 2 years (−65.2 to −41.6; both *p* < 0.001), with the gap widening over time (Table 2c; Figure S2).

### 3.3. Time-Based Analyses

The highest risk was observed during the first 30 days (HR 4.22; 95% CI 3.23–5.53; E-value 7.91) and remained significant at 90 days (HR 2.65), 180 days (HR 2.33), 500 days (HR 1.96), 1000 days (HR 1.84), 1500 days (HR 1.79), 2000 days (HR 1.77), and 2500 days (HR 1.76; all *p* < 0.001) (Table 2).

### 3.4. Sensitivity Analyses

In immortal-time-corrected landmark analyses re-anchoring follow-up beyond the acute ICANS window, the association persisted at the Day 30 landmark (HR 1.30; 95% CI 1.10–1.52) and the Day 90 landmark (HR 1.25; 95% CI 1.05–1.48) (Table 2b). Simpler analyses excluding events within 7, 14, and 21 days were concordant (HR 1.70, 1.43, and 1.33, respectively; all *p* < 0.001; Table S2). The persistence of a significant association beyond the acute window indicates that the elevated risk is not attributable solely to acute ICANS.

### 3.5. Secondary Outcomes

Cognitive Outcomes. CAR-T recipients had a higher risk of overall cognitive impairment (HR 1.58; 95% CI 1.39–1.80; *p* < 0.001), which remained significant after removing altered mental status (R41.82) from the composite (HR 1.61; 95% CI 1.41–1.84), with selective increases in memory impairment (HR 1.38; 95% CI 1.04–1.84; *p* = 0.024) and cognitive dysfunction (HR 1.90; 95% CI 1.37–2.63; *p* < 0.001). Dementia diagnoses were not significantly increased (Table 3).

**Figure 3:**
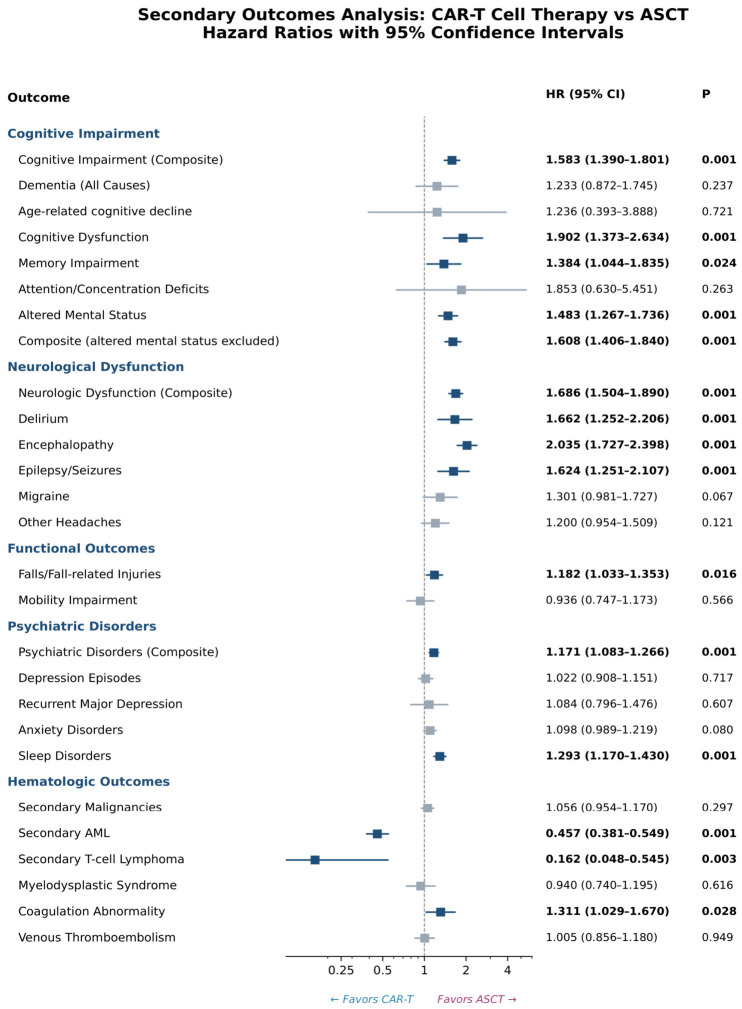
Forest plot of secondary outcomes analysis. Hazard ratios with 95% confidence intervals for secondary outcomes comparing CAR-T versus ASCT. Outcomes include cognitive manifestations, neurological dysfunction, psychiatric disorders, functional outcomes, and hematologic outcomes. CAR-T was associated with increased neurological complications but reduced secondary acute myeloid leukemia risk.

Neurological Dysfunction. Overall neurological dysfunction was 69% higher in the CAR-T group (HR 1.69; 95% CI 1.50–1.89; *p* < 0.001; E-value 2.76). Encephalopathy was the most common complication (HR 2.04; 95% CI 1.73–2.40; *p* < 0.001). Delirium (HR 1.66; 95% CI 1.25–2.21; *p* < 0.001) and seizures (HR 1.62; 95% CI 1.25–2.11; *p* < 0.001) were also significantly more common (Figure 3, Table 3).

Mood and Stress-Related Disorders. Results showed a 17% higher risk in the CAR-T group (HR 1.17; 95% CI 1.08–1.27; *p* < 0.001). Sleep disorders showed the most pronounced difference (HR 1.29; 95% CI 1.17–1.43; *p* < 0.001). The incidence of depressive episodes and anxiety disorders was similar between groups.

Hematologic Outcomes. CAR-T recipients had a significantly lower risk of secondary acute myeloid leukemia (HR 0.46; 95% CI 0.38–0.55; *p* < 0.001; E-value 3.80) and secondary T-cell lymphoma (HR 0.16; 95% CI 0.05–0.55; *p* = 0.003). No significant difference was observed in myelodysplastic syndrome (HR 0.94; *p* = 0.62). Coagulation abnormality was more common (HR 1.31; *p* = 0.028), whereas venous thromboembolism did not differ (HR 1.01; *p* = 0.95) (Table 3, Figure 3).

Functional Outcomes. Fall-related injuries were more common in CAR-T patients (HR 1.18; 95% CI 1.03–1.35; *p* = 0.016). Mobility impairment was not significantly different (HR 0.94; 95% CI 0.75–1.17; *p* = 0.57).

### 3.6. Subgroup Analysis

**Figure 4:**
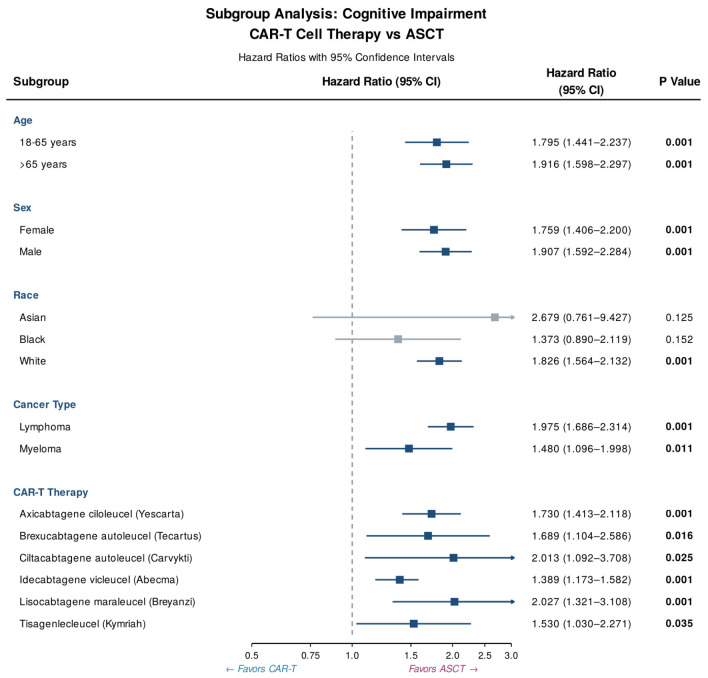
Subgroup analysis of cognitive impairment. Forest plot showing hazard ratios for cognitive impairment across pre-specified subgroups including age, sex, race, cancer type, and CAR-T product. A consistent increased risk was observed across all subgroups, with no significant interaction effects (all *p*-values for interaction > 0.05).

Subgroup hazard ratios remained stable across demographic and clinical categories (Figure 4). The association between CAR-T therapy and cognitive impairment did not differ significantly by age (HR 1.80 for 18–65 years; HR 1.92 for >65 years), gender (HR 1.76 for female; HR 1.91 for male), race, disease type, or CAR-T product type, indicating a consistent association across all subgroups studied.

### 3.7. Negative Control Analysis

To assess specificity and residual bias, we examined negative control outcomes theoretically unrelated to treatment choice. Analyses of gallstones (HR 1.18; 95% CI 0.96–1.46; *p* = 0.12), kidney stones (HR 1.14; 95% CI 0.90–1.45; *p* = 0.27), and appendicitis (HR 0.92; 95% CI 0.50–1.68; *p* = 0.79) showed no significant differences, supporting the validity of the observed significant associations.

## 4. Discussion

This large propensity score-matched study found that CAR-T therapy is associated with a higher risk of long-term cognitive impairment compared with ASCT. The risk was highest in the first month, consistent with acute ICANS. However, even in immortal-time-corrected landmark analyses and when early events were excluded, the increased risk remained throughout follow-up exceeding 2500 days, suggesting both acute and chronic components. Expressed as RMST, CAR-T recipients lost on average roughly 25 and 53 cognitive-impairment-free days over the first 1 and 2 years—an absolute, time-aligned measure that is clinically interpretable in addition to being statistically significant [17,19].

Preclinical studies offer hypotheses that are consistent with these associations: acute ICANS involves cytokine-mediated blood–brain barrier disruption and endothelial activation [25], and animal models show persistent microglial reactivity, oligodendrocyte dysfunction, and impaired hippocampal neurogenesis after CAR-T [26]. However, our study includes no neuroimaging, biomarker, or pathological data and therefore cannot establish a mechanism. The findings should be interpreted as associations; we cannot distinguish chronic CAR-T–specific neurotoxicity from persistent ICANS sequelae, differential detection or surveillance, or survivorship effects. Preclinical reports such as Geraghty et al. [26] are cited only as hypotheses consistent with our observations, not as mechanistic evidence from the present data.

CAR-T products differ in efficacy and toxicity profiles. In our subgroup analysis the association was directionally present for all products, with numerically higher point estimates for ciltacabtagene autoleucel and lisocabtagene maraleucel, although confidence intervals overlapped (Figure 3). Product type is strongly collinear with disease (axicabtagene-, lisocabtagene-, and tisagenlecleucel for lymphoma; idecabtagene- and ciltacabtagene-vicleucel for myeloma; brexucabtagene autoleucel for mantle-cell/ALL), so product-specific effects cannot be fully separated from disease [27,28]. ASCT was selected as an active comparator because it is the most clinically relevant alternative—pivotal trials (ZUMA-7, TRANSFORM) compared CAR-T against salvage chemotherapy followed by ASCT—and because an active comparator reduces confounding by indication relative to an untreated reference [4,5]. Nonetheless, channeling bias is possible: in the CAR-T era, CAR-T is often used after ASCT failure or as second-line therapy, so the two groups may differ in ways residual to matching. Restricting ASCT to the contemporaneous era partly mitigates, but does not eliminate, this concern [16].

Our study found lower rates of secondary acute myeloid leukemia in CAR-T recipients compared with ASCT recipients, likely reflecting differences in conditioning regimens. These findings provide some reassurance regarding the hematologic safety of CAR-T, although continued follow-up for late events is needed [29].

### Limitations

Limitations include reliance on diagnostic codes rather than formal neuropsychological testing (e.g., MoCA, MMSE, or the ICCTF cognitive battery), which may misclassify cognitive outcomes; because ascertainment was identical in both arms within the same network, such misclassification is expected to be non-differential and to bias estimates toward the null, implying the true association is at least as large as observed. The platform did not permit a confirmed-diagnosis definition requiring repeated, temporally separated coding, which would further enhance specificity [30]. Important confounders that TriNetX cannot capture—education and cognitive reserve, socioeconomic status, treatment preference, and performance status—were unmeasured; we quantified robustness to such unmeasured confounding using E-values [23]. Other limitations include potential residual confounding despite excellent post-matching balance and shorter follow-up in the CAR-T cohort, which we partly addressed with time-aligned RMST and landmark analyses. We also note that the study was not prospectively registered before data extraction; the protocol has been retrospectively deposited on the Open Science Framework as a transparency measure, but retrospective deposition does not provide the same safeguard against outcome switching or selective reporting that prospective registration would. Nevertheless, the large sample, rigorous methodology, negative-control outcomes, and time-stratified analyses enhance validity.

## 5. Conclusions

CAR-T therapy is associated with a higher risk of cognitive impairment that extends well beyond the acute phase. Because these are observational, code-based associations rather than mechanistic findings, they highlight the need for prospective studies with standardized cognitive testing, biomarker collection, and interventional trials targeting neuroinflammation to reduce long-term cognitive risks in CAR-T recipients [26,31].

## Figures and Tables

**Table 1:** Patient Characteristics Before and After Propensity Score Matching.

Characteristic	Before PSM				After PSM			
	CAR-T (*n* = 3096)	ASCT (*n* = 34,734)	*p*	SMD	CAR-T (*n* = 3067)	ASCT (*n* = 3067)	*p*	SMD
Demographics								
Age at index, mean (SD), y	60.5 (16.0)	57.7 (15.1)	<0.001	0.180	60.1 (16.2)	59.8 (13.2)	0.427	0.020
Male	1932 (62.4)	20,151 (58.0)	<0.001	0.090	1914 (62.4)	1920 (62.6)	0.874	0.004
Female	1164 (37.6)	14,577 (42.0)	<0.001	0.089	1153 (37.6)	1147 (37.4)	0.874	0.004
Race								
White	2421 (78.2)	25,474 (73.3)	<0.001	0.114	2377 (77.5)	2343 (76.4)	0.303	0.026
Black or African American	334 (10.8)	4852 (14.0)	<0.001	0.097	344 (11.2)	347 (11.3)	0.904	0.003
Asian	84 (2.7)	759 (2.2)	0.056	0.034	83 (2.7)	83 (2.7)	1.000	<0.001
Other race	118 (3.8)	1690 (4.9)	0.008	0.052	122 (4.0)	125 (4.1)	0.846	0.005
Unknown race	139 (4.5)	1959 (5.6)	0.007	0.052	141 (4.6)	169 (5.5)	0.103	0.042
Primary Diagnosis								
Non-follicular lymphoma (DLBCL)	1802 (58.2)	5560 (16.0)	<0.001	0.971	1662 (54.2)	1681 (54.8)	0.626	0.012
Follicular lymphoma	409 (13.2)	1313 (3.8)	<0.001	0.343	371 (12.1)	349 (11.4)	0.383	0.022
Multiple myeloma	1015 (32.8)	20,841 (60.0)	<0.001	0.567	1034 (33.7)	1037 (33.8)	0.935	0.002
Comorbidities								
Diabetes mellitus	598 (19.3)	6110 (17.6)	0.016	0.044	592 (19.3)	570 (18.6)	0.473	0.018
Hypertensive diseases	1573 (50.8)	16,267 (46.8)	<0.001	0.080	1543 (50.3)	1524 (49.7)	0.628	0.012
Ischemic heart disease	523 (16.9)	4263 (12.3)	<0.001	0.131	491 (16.0)	491 (16.0)	1.000	<0.001
Heart failure	334 (10.8)	3058 (8.8)	<0.001	0.067	319 (10.4)	304 (9.9)	0.526	0.016
Chronic kidney disease	455 (14.7)	5393 (15.5)	0.221	0.023	448 (14.6)	439 (14.3)	0.744	0.008
Cerebral infarction	87 (2.8)	550 (1.6)	<0.001	0.084	80 (2.6)	77 (2.5)	0.808	0.006
Other rheumatoid arthritis	40 (1.3)	455 (1.3)	0.933	0.002	37 (1.2)	55 (1.8)	0.059	0.048
Systemic lupus erythematosus	9 (0.3)	162 (0.5)	0.163	0.029	9 (0.3)	9 (0.3)	1.000	<0.001
Mental Health								
Mood disorders	573 (18.5)	5473 (15.8)	<0.001	0.073	561 (18.3)	567 (18.5)	0.843	0.005
Anxiety disorders	898 (29.0)	8074 (23.2)	<0.001	0.131	874 (28.5)	846 (27.6)	0.426	0.020
Cognitive symptoms (R41)	353 (11.4)	2026 (5.8)	<0.001	0.199	344 (11.2)	334 (10.9)	0.684	0.010
Schizophrenia & psychotic disorders	19 (0.6)	158 (0.5)	0.215	0.022	18 (0.6)	15 (0.5)	0.601	0.013
Procedure/Treatment								
Intrathecal chemotherapy	325 (10.5)	1680 (4.8)	<0.001	0.214	313 (10.2)	319 (10.4)	0.801	0.006
Antineoplastic radiation therapy	195 (6.3)	917 (2.6)	<0.001	0.178	166 (5.4)	153 (5.0)	0.455	0.019
Medication								
Corticosteroids (systemic)	2619 (84.6)	23,210 (66.8)	<0.001	0.424	2567 (83.7)	2635 (85.9)	0.016	0.062
Rituximab	687 (22.2)	1786 (5.1)	<0.001	0.512	567 (18.5)	592 (19.3)	0.415	0.021
Bendamustine	307 (9.9)	353 (1.0)	<0.001	0.399	187 (6.1)	169 (5.5)	0.326	0.025
Daratumumab	238 (7.7)	2464 (7.1)	0.219	0.023	248 (8.1)	245 (8.0)	0.888	0.004
Lenalidomide	229 (7.4)	7230 (20.8)	<0.001	0.393	239 (7.8)	218 (7.1)	0.307	0.026
Carfilzomib	217 (7.0)	807 (2.3)	<0.001	0.224	218 (7.1)	215 (7.0)	0.881	0.004
Bortezomib	121 (3.9)	4537 (13.1)	<0.001	0.333	129 (4.2)	120 (3.9)	0.560	0.015
Polatuzumab vedotin	211 (6.8)	58 (0.2)	<0.001	0.368	83 (2.7)	55 (1.8)	0.016	0.062
Brentuximab vedotin	9 (0.3)	431 (1.2)	<0.001	0.109	9 (0.3)	9 (0.3)	1.000	<0.001
Laboratory Values								
BMI, mean (SD), kg/m^2^	28.4 (6.3)	28.9 (6.5)	<0.001	0.078	28.4 (6.4)	28.7 (6.1)	0.060	0.048
LDH, *n* (%)								
LDH ≤ 250 U/L	1484 (47.9)	18,928 (54.5)	<0.001	0.132	1488 (48.5)	1537 (50.1)	0.211	0.032
LDH 251–500 U/L	1077 (34.8)	11,337 (32.6)	0.015	0.045	1063 (34.7)	1044 (34.0)	0.609	0.013
LDH ≥ 500 U/L	535 (17.3)	4469 (12.9)	<0.001	0.124	516 (16.8)	486 (15.8)	0.300	0.026

**Notes:** SMD < 0.10 indicates adequate balance between groups after propensity score matching. *p* values from *t*-test (continuous) or chi-square test (categorical). Race categories (White, Black or African American, Asian, Other race, Unknown race) are mutually exclusive and sum to the cohort total; “Other race” comprises American Indian or Alaska Native, Native Hawaiian or Other Pacific Islander, and other recorded categories. Primary diagnosis groups are mutually exclusive. **Abbreviations:** ASCT, autologous stem cell transplantation; BMI, body mass index; CAR-T, chimeric antigen receptor T-cell therapy; DLBCL, diffuse large B-cell lymphoma; LDH, lactate dehydrogenase; PSM, propensity score matching; SD, standard deviation; SMD, standardized mean difference.

**Table 2:** Time Course and Magnitude of Cognitive Impairment Risk After CAR-T Cell Therapy versus Autologous Stem Cell Transplantation. (**a**) Kaplan–Meier Cumulative Incidence of Cognitive Impairment at Fixed Time Points. CAR-T cell therapy versus autologous stem cell transplantation. Cumulative incidence estimated as 1−Kaplan–Meier survival probability; (**b**) Sensitivity Analyses Using Landmark Models to Exclude the Acute ICANS Window (Day 30 and Day 90 Landmarks); (**c**) Restricted Mean Survival Time (RMST) for Cognitive-Impairment-Free Survival.

**(a)**					
**Time From** **Treatment**	**CAR-T Cumulative** **Incidence, % (95% CI)**	**ASCT Cumulative** **Incidence, % (95% CI)**	**Risk Difference, %** **(CAR-T − ASCT)**	**Risk Ratio** **(95% CI)**	**Cumulative HR** **(95% CI)**
30 days	9.8 (8.8, 10.9)	2.9 (2.4, 3.6)	6.9 (5.7, 8.1)	3.35 (2.67, 4.22)	4.22 (3.23, 5.53)
90 days	11.9 (10.9, 13.1)	5.1 (4.3, 5.9)	6.9 (5.5, 8.3)	2.36 (1.97, 2.83)	2.65 (2.16, 3.25)
180 days	13.7 (12.5, 15.0)	6.9 (6.1, 7.9)	6.8 (5.2, 8.3)	1.97 (1.69, 2.31)	2.33 (1.94, 2.79)
500 days	17.9 (16.5, 19.4)	10.4 (9.4, 11.6)	7.5 (5.6, 9.3)	1.72 (1.50, 1.97)	1.96 (1.68, 2.28)
1000 days	21.3 (19.6, 23.2)	14.5 (13.2, 16.0)	6.8 (4.5, 9.1)	1.47 (1.29, 1.67)	1.84 (1.60, 2.13)
1500 days	23.3 (21.2, 25.5)	17.9 (16.3, 19.6)	5.4 (2.7, 8.1)	1.30 (1.14, 1.48)	1.79 (1.56, 2.06)
2000 days	24.8 (22.2, 27.6)	20.7 (18.9, 22.6)	4.1 (0.8, 7.3)	1.20 (1.04, 1.38)	1.77 (1.54, 2.03)
2500 days †	24.8 (22.2, 27.6)	23.9 (21.8, 26.2)	0.8 (−2.6, 4.3)	1.03 (0.90, 1.19)	1.76 (1.54, 2.02)
Overall, full follow-up	Censoring-adjusted Cox summary over the entire follow-up. The crude proportion (CAR-T 16.6% vs. ASCT 16.2%, RR 1.02) is not reported, as it ignores the differential follow-up and censoring between the cohorts.				1.58 (1.39–1.80); log-rank *p* < 0.001
**(b)**					
**Sensitivity Analysis**	**Patients at Risk at** **Landmark, CAR-T/ASCT**		**HR (95% CI)**		***p* Value**
Primary analysis (from index date)	3067/3067		1.58 (1.39–1.80)		<0.001
Day 30 landmark	2800/3000		1.30 (1.10–1.52)		<0.01
Day 90 landmark	2710/2900		1.25 (1.05–1.48)		<0.05
**(c)**					
**Time Horizon**	**CAR-T RMST,** **Days (95% CI)**		**ASCT RMST,** **Days (95% CI)**	**RMST Difference,** **Days (95% CI)**	***p***
1 year (365 days)	316.1 (311.8–320.4)		340.9 (338.0–343.9)	−24.8 (−30.1, −19.6)	<0.001
2 years (730 days)	612.2 (602.6–621.7)		665.6 (658.7–672.5)	−53.4 (−65.2, −41.6)	<0.001
3 years (1095 days)	898.5 (882.8–914.3)		978.7 (967.3–990.1)	−80.2 (−99.6, −60.7)	<0.001

**Number at risk (**Figure 2**):** CAR-T—0 d 3067; 500 d 839; 1000 d 454; 1500 d 187; 2000 d 54; 2500 d 0. ASCT—0 d 3067; 500 d 1882; 1000 d 1560; 1500 d 1226; 2000 d 828; 2500 d 255. † At ≥2000 days the CAR-T cohort retains very few patients at risk (54 at 2000 days; 0 by 2500 days); estimates at the late tail are unstable, and the apparent convergence reflects exhaustion of CAR-T follow-up rather than equalized risk. Interpretation should focus on the first ~2 years, where the between-group difference is large and statistically significant. **Cumulative HR** is the Cox hazard ratio estimated from the index date to each landmark. The monotonic decline in the cumulative HR (from 4.22 at 30 days to 1.76 at 2500 days), mirrored by the time-aligned risk ratio, indicates that the proportional-hazards assumption is not satisfied; the hazard ratios are therefore presented across time intervals rather than as a single summary estimate. **Notes:** Cumulative incidence and its 95% CI are taken directly from the Kaplan–Meier estimator at each landmark; the risk difference is the between-group difference in cumulative incidence (95% CI by the normal approximation) and the risk ratio is the ratio of cumulative incidences (95% CI by the delta method on the log scale). Because all estimates are time-aligned and account for censoring, the risk ratio and the cumulative incidence are mutually consistent at every time point. **Day 30/Day 90 landmark:** follow-up time is re-anchored at the landmark; only patients alive, in follow-up, and free of cognitive impairment at the landmark contribute subsequent person-time. **Notes:** RMST is the average number of days free of cognitive impairment within each time horizon, estimated as the area under the Kaplan–Meier impairment-free survival curve from the index date to the horizon. A negative difference indicates fewer impairment-free days in the CAR-T group relative to ASCT. Because the proportional-hazards assumption was violated (Schoenfeld global test, *p* < 0.001), RMST is presented as the principal, assumption-free effect measure; the single Cox hazard ratio (1.58) is retained only as a time-averaged summary, and time-stratified hazard ratios (Table 2a) and Kaplan–Meier curves (Figure 2) are retained for interpretation. RMST and its variance were derived from the Kaplan–Meier estimates using the standard variance estimator; the patient-level risk sets required for the variance were reconstructed from the exported Kaplan–Meier coordinates and numbers-at-risk by the method of Guyot et al. **Abbreviations:** ASCT, autologous stem cell transplantation; CAR-T, chimeric antigen receptor T-cell therapy; CI, confidence interval; HR, hazard ratio; ICANS, immune cell-associated neurotoxicity syndrome; RMST, restricted mean survival time; RR, risk ratio [1].

**Table 3:** Secondary Outcomes Analysis (Kaplan–Meier Cumulative Incidence).

Outcome	CAR-T Cumulative Incidence, % (95% CI)	ASCT Cumulative Incidence, % (95% CI)	Risk Ratio (95% CI)	Hazard Ratio (95% CI)	*p*	E-Value (Point [CI])
**Cognitive Impairment**						
Composite	24.8 (22.2–27.6)	23.0 (21.5–24.6)	**1.351 (1.184–1.541)**	**1.583 (1.390–1.801)**	**<0.001**	2.54 (2.13)
Dementia (All Causes)	2.3 (1.6–3.3)	3.0 (2.4–3.9)	0.766 (0.498–1.180)	1.233 (0.872–1.745)	0.237	1.77 (1.00)
Age-related cognitive decline ^#^	—	—	—	1.236 (0.393–3.888)	0.721	1.78 (1.00)
Cognitive Dysfunction	4.4 (3.2–5.9)	3.5 (2.8–4.4)	1.241 (0.847–1.820)	**1.902 (1.373–2.634)**	**<0.001**	3.21 (2.09)
Memory Impairment	4.7 (3.6–6.3)	4.7 (3.9–5.8)	0.999 (0.706–1.414)	**1.384 (1.044–1.835)**	**0.024**	2.11 (1.26)
Attention/Concentration Deficits ^#^	—	—	—	1.853 (0.630–5.451)	0.263	3.11 (1.00)
Altered Mental Status	16.9 (15.2–18.7)	10.9 (9.6–12.3)	**1.552 (1.324–1.819)**	**1.483 (1.267–1.736)**	**<0.001**	2.33 (1.85)
Composite (excluding altered mental status)	8.0 (6.6–9.8)	7.3 (6.3–8.5)	**1.201 (1.006–1.396)**	**1.608 (1.406–1.840)**	**<0.001**	2.60 (2.16)
**Neurological Dysfunction**						
Composite	26.0 (23.9–28.3)	19.5 (18.0–21.2)	**1.331 (1.183–1.498)**	**1.686 (1.504–1.890)**	**<0.001**	2.76 (2.37)
Delirium	4.5 (3.4–5.9)	2.9 (2.3–3.7)	**1.571 (1.087–2.272)**	**1.662 (1.252–2.206)**	**<0.001**	2.71 (1.81)
Encephalopathy	14.7 (13.2–16.5)	9.2 (8.1–10.4)	**1.608 (1.354–1.908)**	**2.035 (1.727–2.398)**	**<0.001**	3.49 (2.85)
Epilepsy/Seizures	6.1 (5.1–7.3)	4.9 (4.1–5.8)	1.247 (0.965–1.612)	**1.624 (1.251–2.107)**	**<0.001**	2.63 (1.81)
Migraine	3.9 (3.0–5.1)	4.1 (3.4–5.1)	0.945 (0.676–1.321)	1.301 (0.981–1.727)	0.067	1.93 (1.00)
Other Headaches	5.5 (4.4–7.0)	4.8 (4.0–5.9)	1.141 (0.840–1.551)	1.200 (0.954–1.509)	0.121	1.69 (1.00)
**Functional Outcomes**						
Falls/Fall-related Injuries	23.5 (20.8–26.3)	20.5 (18.8–22.3)	1.144 (0.990–1.322)	**1.182 (1.033–1.353)**	**0.016**	1.65 (1.22)
Mobility Impairment ^#^	—	—	—	0.936 (0.747–1.173)	0.566	1.34 (1.00)
**Psychiatric Disorders**						
Composite	51.4 (48.7–54.1)	50.9 (48.9–53.0)	1.010 (0.945–1.079)	**1.171 (1.083–1.266)**	**<0.001**	1.62 (1.38)
Depression Episodes	22.8 (20.6–25.2)	23.9 (22.2–25.7)	0.955 (0.844–1.080)	1.022 (0.908–1.151)	0.717	1.17 (1.00)
Recurrent Major Depression	4.1 (3.1–5.4)	5.0 (4.2–6.0)	0.819 (0.585–1.146)	1.084 (0.796–1.476)	0.607	1.39 (1.00)
Anxiety Disorders	30.0 (27.7–32.5)	29.5 (27.7–31.5)	1.015 (0.917–1.124)	1.098 (0.989–1.219)	0.080	1.43 (1.00)
Sleep Disorders	32.6 (30.1–35.3)	31.2 (29.3–33.1)	1.046 (0.947–1.156)	**1.293 (1.170–1.430)**	**<** **0.001**	1.91 (1.62)
**Hematologic Outcomes**						
Secondary Malignancies	32.5 (30.1–35.1)	32.1 (30.1–34.1)	1.015 (0.919–1.121)	1.056 (0.954–1.170)	0.297	1.30 (1.00)
Secondary AML	7.7 (6.4–9.2)	14.7 (13.3–16.2)	**0.524 (0.428–0.642)**	**0.457 (0.381–0.549)**	**<0.001**	3.80 (3.04)
Secondary T-cell Lymphoma ^#^	—	—	—	**0.162 (0.048–0.545)**	**0.003**	11.82 (3.07)
Myelodysplastic Syndrome	5.8 (4.5–7.3)	6.5 (5.5–7.6)	0.889 (0.665–1.188)	0.940 (0.740–1.195)	0.616	1.32 (1.00)
Coagulation Abnormality	6.9 (5.6–8.4)	3.9 (3.2–4.8)	**1.749 (1.305–2.344)**	**1.311 (1.029–1.670)**	**0.028**	1.95 (1.20)
Venous Thromboembolism	13.8 (12.0–15.8)	11.2 (10.0–12.6)	**1.233 (1.029–1.477)**	1.005 (0.856–1.180)	0.949	1.08 (1.00)
**Negative Control Outcomes**						
Gallstones	8.8 (7.3–10.7)	7.5 (6.6–8.7)	1.148 (0.749–1.733)	1.183 (0.959–1.461)	0.116	1.65 (1.00)
Kidney Stones	6.4 (5.2–7.8)	6.2 (5.2–7.3)	1.026 (0.785–1.341)	1.142 (0.901–1.449)	0.271	1.54 (1.00)
Appendicitis	0.8 (0.5–1.3)	0.8 (0.5–1.2)	1.001 (0.491–2.040)	0.920 (0.503–1.683)	0.785	1.39 (1.00)

**Notes:** Cumulative incidence was estimated as 1−Kaplan–Meier survival probability, with 95% confidence intervals from the Kaplan–Meier estimator; the risk ratio is the ratio of the two cumulative incidences (95% CI by the delta method on the log scale). Hazard ratios, *p*-values, and E-values are derived from the platform’s Cox survival analysis over the full follow-up. Because the cumulative risk ratio and the hazard ratio are estimated over different time frames, modest differences between them are expected, particularly where hazards are non-proportional. Bold denotes *p* < 0.05 (hazard ratio) or a risk ratio whose 95% CI excludes 1. “Composite (excluding altered mental status)” is a sensitivity analysis for the primary cognitive composite with altered mental status (R41.82) removed. Negative-control outcomes (gallstones, kidney stones, appendicitis) are not expected to differ between groups and showed risk ratios near 1, supporting the absence of systematic bias. E-values are reported for the point estimate and, in parentheses, for the confidence-interval limit closest to the null; a value of 1.00 indicates a 95% CI that already includes the null. All outcomes are reported for the propensity score–matched cohort (*n* = 3067 per group). **^#^** Per TriNetX privacy policy, results are not reported when a cell count is below 10; Kaplan–Meier cumulative incidence could therefore not be estimated for these outcomes, and only the hazard ratio from the platform’s survival analysis is shown. **Abbreviations:** AML, acute myeloid leukemia; ASCT, autologous stem cell transplantation; CAR-T, chimeric antigen receptor T-cell therapy; CI, confidence interval.

## Data Availability

The data presented in this study are available from the TriNetX Research Network (https://trinetx.com, accessed on 14 June 2026). Access to TriNetX data requires institutional membership and compliance with the network’s data use agreements. Summary data supporting the findings of this study are available from the corresponding authors upon reasonable request.

## Supplementary Materials

The following supporting information can be downloaded at: https://www.mdpi.com/article/10.3390/diagnostics16121862/s1, STROBE Checklist; Supplementary Methods (S1–S6), S1. Data Source and Study Population, S2. Detailed Cohort Definitions, S3. Outcome Definitions and ICD-10 Codes, S4. Propensity Score Matching Methodology, S5. Statistical Analysis Plan, S6. Sensitivity, Robustness, and Negative-Control Analyses; Figure S1: Graphical representation of study design; Figure S2: Restricted Mean Survival Time (RMST) for Cognitive-Impairment-Free Survival; Table S1: Complete ICD-10 code definitions for all outcomes; Table S2: Sensitivity Analyses Excluding Events Within the Acute ICANS Windows.
