# Supplementary material for: Long-Term Cognitive Impairment After CAR-T Therapy Versus Autologous Stem Cell Transplantation: A Propensity Score-Matched Cohort Study
Source: Diagnostics (Basel). 2026 Jun 16;16(12):1862. doi: 10.3390/diagnostics16121862 (PMC13298195; doi:10.3390/diagnostics16121862)
Supplement: Supplementary file 1 [file diagnostics-16-01862-s001.zip › diagnostics-4325333-supplementary.pdf]

Supplementary Materials

Long-Term Cognitive Impairment After CAR-T Therapy Versus Autologous Stem Cell Transplantation: A Propensity Score-Matched Cohort Study

*Anna Byzniuk, Po-Huang Chen, Wei-Cheng Chang, Hsin-Yu Chen, Li-Ting Kao, Tina Yi-Jin Hsieh, Ming-Shen Dai, Hong-Jie Jhou * and Cho-Hao Lee **

Table of Contents

STROBE Checklist
Supplementary Methods
S1. Data Source and Study Population
S2. Detailed Cohort Definitions
S3. Outcome Definitions and ICD-10 Codes
S4. Propensity Score Matching Methodology
S5. Statistical Analysis Plan
S6. Sensitivity, Robustness, and Negative-Control Analyses
Supplementary Figures
Figure S1. Graphical Representation of Study Design
Figure S2. Restricted Mean Survival Time (RMST) for Cognitive-Impairment–Free Survival
Supplementary Tables
Table S1. Complete ICD-10 Code Definitions for All Outcomes
Table S2. Sensitivity Analyses Excluding Events Within the Acute ICANS Window

STROBE Checklist

Strengthening the Reporting of Observational Studies in Epidemiology

Item	No	Recommendation	Page / Location
**Title and Abstract**			
Title and abstract	1	(a) Indicate the study's design with a commonly used term in the title or abstract; (b) Provide an informative and balanced summary of what was done and what was found	Title page; Abstract
**Introduction**			
Background/rationale	2	Explain the scientific background and rationale for the investigation being reported	Introduction, paragraphs 1–2
Objectives	3	State specific objectives, including any prespecified hypotheses	Introduction, paragraph 3
**Materials and Methods**			
Study design	4	Present key elements of study design early in the paper	Section 2.1
Setting	5	Describe the setting, locations and relevant dates, including periods of recruitment, exposure, follow-up, and data collection	Section 2.1; Supplementary S1, S2
Participants	6	(a) Give the eligibility criteria, sources, and methods of selection; describe methods of follow-up; (b) for matched studies, give matching criteria and numbers of exposed and unexposed	Sections 2.2, 2.3, 2.5; Supplementary S2, S4
Variables	7	Clearly define all outcomes, exposures, predictors, potential confounders, and effect modifiers; give diagnostic criteria	Section 2.4; Supplementary S3 (Table S1)
Data sources/measurement	8	For each variable of interest, give sources of data and details of methods of assessment	Section 2.2; Supplementary S1, S3
Bias	9	Describe any efforts to address potential sources of bias	Sections 2.5, 2.6; Supplementary S4, S6; negative-control outcomes (Table 3)
Study size	10	Explain how the study size was arrived at	Section 2.2; Figure 1
Quantitative variables	11	Explain how quantitative variables were handled in the analyses	Section 2.6; Supplementary S4, S5
Statistical methods	12	(a) Methods to control for confounding; (b) subgroups and interactions; (c) missing data; (d) loss to follow-up; (e) sensitivity analyses, including the proportional-hazards test and restricted mean survival time	Section 2.6; Supplementary S4–S6; Tables 2a–2c; Table S2; Figure S2
**Results**			
Participants	13	(a) Numbers at each stage; (b) reasons for non-participation; (c) flow diagram	Section 3.1; Figure 1
Descriptive data	14	(a) Characteristics of participants; (b) missing data; (c) follow-up time	Section 3.1; Table 1
Outcome data	15	Report numbers of outcome events or summary measures over time	Sections 3.2–3.3; Tables 2a–2c; Figure 2
Main results	16	(a) Unadjusted and adjusted estimates with precision; (b) category boundaries; (c) translate relative risk into absolute risk where relevant	Tables 2a–2c, Table 3; Section 3.6
Other analyses	17	Report subgroup, interaction, and sensitivity analyses	Sections 3.4, 3.6, 3.7;

			Tables 2a–2c; Figure 3; Table 3; Table S2; Figure S2
**Discussion**			
Key results	18	Summarise key results with reference to study objectives	Discussion, paragraph 1
Limitations	19	Discuss limitations, considering sources of bias or imprecision	Section 4.1
Interpretation	20	Cautious overall interpretation considering objectives, limitations, multiplicity of analyses, and similar studies	Discussion, paragraphs 2–4
Generalisability	21	Discuss generalisability (external validity)	Section 4.1
**Other Information**			
Funding	22	Source of funding and the role of the funders for the present study	Funding section

Reference: *von Elm E, Altman DG, Egger M, Pocock SJ, Gøtzsche PC, Vandenbroucke JP. The Strengthening the Reporting of Observational Studies in Epidemiology (STROBE) statement. Lancet 2007;370:1453–1457.*

Supplementary Methods

S1. Data Source and Study Population

This retrospective cohort study used the TriNetX US Collaborative Network, a federated health-research platform that aggregates electronic health records from more than 100 healthcare organizations, including academic medical centers, community hospitals, and specialty clinics. TriNetX provides longitudinal patient data—demographics, diagnoses, procedures, medications, and laboratory results—while maintaining privacy through statistical de-identification compliant with the Health Insurance Portability and Accountability Act (HIPAA).

All clinical data are coded using standardized terminologies: ICD-10-CM for diagnoses; CPT and HCPCS for procedures; RxNorm for medications; and LOINC for laboratory tests. The study period extended from 1 January 2014 through 1 April 2025 (database extraction date 1 April 2025), capturing CAR-T adoption since the first FDA approval (Kymriah, August 2017) with sufficient follow-up for outcome assessment.

S2. Detailed Cohort Definitions

S2.1. CAR-T Therapy Cohort

Patients were identified using drug names with RxNorm and HCPCS codes:

- Kymriah (tisagenlecleucel): RxNorm 1946831 — FDA approved August 2017
- Yescarta (axicabtagene ciloleucel): RxNorm 1946834 — FDA approved October 2017
- Tecartus (brexucabtagene autoleucel): RxNorm 2379937; HCPCS Q2053 — FDA approved July 2020
- Breyanzi (lisocabtagene maraleucel): RxNorm 2379934; HCPCS Q2054 — FDA approved February 2021
- Abecma (idecabtagene vicleucel): RxNorm 2470735; HCPCS Q2055 — FDA approved March 2021
- Carvykti (ciltacabtagene autoleucel): RxNorm 2470738; HCPCS Q2056 — FDA approved February 2022

The index date was the date of CAR-T infusion.

S2.2. ASCT Cohort

Patients were identified using CPT 38241 (autologous hematopoietic progenitor cell transplantation) and ICD-10-PCS 30233G0 (introduction of autologous hematopoietic stem or progenitor cells into a peripheral vein, percutaneous), with a confirmed diagnosis of lymphoma or multiple myeloma. The index date was the date of stem cell infusion. **To ensure a contemporaneous comparison and to address calendar-era differences in supportive care, coding practice, and patient selection, the ASCT cohort was restricted to an index date on or after August 2017 (the first FDA CAR-T approval); ASCT recipients with an earlier index date were excluded (Figure 1).**

S2.3. Exclusion Criteria

- Age <18 years at index date (the pediatric population has distinct treatment protocols);
- Cognitive disorder diagnosis within 1 year prior to index date;
- Receipt of both CAR-T and ASCT during the study period;
- ASCT index date before August 2017 (contemporaneous-comparison restriction);
- Insufficient follow-up (<30 days after the index date).

S3. Outcome Definitions and ICD-10 Codes

The primary composite outcome of cognitive impairment comprised the first post-index occurrence of any constituent code (Table S1). **A prespecified sensitivity definition removed R41.82 (altered mental status)—the least specific component, which overlaps with acute/ICANS-related presentations—to test specificity for chronic cognitive impairment.** Secondary outcomes included neurological dysfunction (delirium, encephalopathy, seizures, headache/migraine), mood and stress-related disorders (depression, anxiety, sleep disorders), functional outcomes (falls, mobility impairment), hematologic outcomes (secondary acute myeloid leukemia, myelodysplastic syndrome, secondary T-cell lymphoma, coagulation abnormality, venous thromboembolism), and negative-control outcomes. Complete ICD-10 definitions are provided in Table S1.

S4. Propensity Score Matching Methodology

Propensity scores were estimated by logistic regression modeling the probability of receiving CAR-T versus ASCT conditional on the following baseline covariates:

- Demographics: age at index (continuous), sex, race (White, Black or African American, Asian, Other, Unknown)
- Primary cancer diagnosis: non-follicular lymphoma (DLBCL), follicular lymphoma, multiple myeloma
- CNS involvement / CNS-directed therapy: secondary malignant neoplasm of brain, intrathecal chemotherapy, antineoplastic radiation therapy
- Comorbidities: diabetes mellitus, hypertensive diseases, ischemic heart disease, heart failure, chronic kidney disease, cerebral infarction, rheumatoid arthritis, systemic lupus erythematosus
- Mental-health conditions: mood disorders, anxiety disorders, schizophrenia-spectrum disorders, baseline cognitive symptoms (R41)
- **Prior / concomitant disease-directed treatment:** systemic corticosteroids, rituximab, bendamustine, daratumumab, lenalidomide, carfilzomib, bortezomib, polatuzumab vedotin, brentuximab vedotin
- Laboratory values: body mass index; lactate dehydrogenase (LDH), **modeled as a categorical variable (≤250, 251–500, ≥500 U/L).**

LDH was categorized rather than entered as a continuous covariate because extreme values produced residual imbalance (continuous LDH standardized mean difference 0.141 after matching). Categorization anchored to clinical thresholds (approximately the upper limit of normal and twice the upper limit of normal) removed the outlier leverage and achieved balance (all category standardized mean differences <0.05), while preserving prognostic information relevant to tumor burden.

Matching Algorithm

- 1:1 nearest-neighbor matching without replacement
- Caliper: 0.2 × the standard deviation of the logit of the propensity score
- Balance assessed by standardized mean differences (SMD <0.10 considered adequate)

S5. Statistical Analysis Plan

Descriptive Statistics

Continuous variables are summarized as mean (SD) or median (IQR); categorical variables as frequencies and percentages. P values are from t-tests (continuous) or chi-square tests (categorical). Median follow-up was 634 days (CAR-T) and 713 days (ASCT).

Time-to-Event Analysis

Cumulative incidence was estimated as 1 − Kaplan–Meier survival and compared by log-rank test; Cox proportional-hazards models provided hazard ratios (HR) with 95% CIs. To characterize the time course, cumulative incidence and the cumulative (index-to-landmark) hazard ratio were reported at fixed landmarks (30, 90, 180, 500, 1,000, 1,500, 2,000, and 2,500 days; Table 2a).

Proportional-Hazards Assessment and Principal Effect Measure

The proportional-hazards assumption was evaluated using the Schoenfeld residual global test and was violated (P<0.001), consistent with the monotonic decline in the cumulative hazard ratio over time. **The single Cox hazard ratio was therefore retained only as a time-averaged summary, and the principal, assumption-free effect measure was the restricted mean survival time (RMST) difference—the difference in mean cognitive-impairment–free days—at 1, 2, and 3 years (Table 2c).** RMST and its variance were derived from the Kaplan–Meier estimates using the standard variance estimator; the patient-level risk sets required for the variance were reconstructed from the exported Kaplan–Meier coordinates and numbers-at-risk by the method of Guyot et al. Time-stratified hazard ratios (Table 2a) and Kaplan–Meier curves (Figure 2) were retained for interpretation.

Landmark Analyses

To address delayed or prolonged ICANS, landmark analyses re-anchored follow-up at Day 30 (primary landmark) and Day 90, including only patients alive, in follow-up, and free of cognitive impairment at the landmark (Table 2b). Simpler analyses excluding events within 7, 14, and 21 days are reported in Table S2.

Subgroup Analyses
Prespecified subgroups were age (18–65 vs >65 years), sex, race, disease type (lymphoma vs myeloma), and CAR-T product; interactions were tested by likelihood-ratio tests (Figure 3).

Software
Cohort construction, matching, Kaplan–Meier, and Cox analyses were performed in TriNetX Analytics; the Schoenfeld test and RMST were computed in R (survival, survRM2).

S6. Sensitivity, Robustness, and Negative-Control Analyses

Sensitivity Analyses (Acute ICANS Window)
Because acute ICANS typically occurs within the first 30 days after CAR-T infusion, two complementary strategies isolated chronic effects from acute neurotoxicity. First, immortal-time–corrected landmark analyses re-anchored follow-up at Day 30 and Day 90 (Table 2b). Second, analyses excluded cognitive-impairment events occurring within 7, 14, and 21 days after the index date (Table S2). The association remained statistically significant across all windows, indicating that the elevated risk is not attributable solely to acute ICANS.

E-Value Analysis
E-values were calculated for primary and secondary outcomes to quantify the minimum strength of association an unmeasured confounder would need with both treatment and outcome to fully explain the observed association (interpretation: <1.5 weak, 1.5–2.5 moderate, 2.5–4.0 strong, >4.0 very strong robustness).

Negative-Control Analyses
Outcomes theoretically unrelated to the CAR-T versus ASCT choice—gallstones/cholelithiasis (K80), kidney stones/nephrolithiasis (N20.0–N20.2), and appendicitis (K35–K37)—were examined. These conditions were chosen because they are: (i) common enough in this oncology population (incidence approximately 5–10%) to provide statistical power; (ii) clinically and biologically unrelated to the cognitive and neurological outcomes of interest, with no plausible shared inflammatory, cytotoxic, or neuro-immune pathway with either CAR-T or ASCT; and (iii) typically diagnosed through acute symptomatic presentation and imaging rather than through active surveillance, so that equal incidence between arms reflects equivalent access to care and equivalent coding intensity rather than equivalent screening. Hazard ratios near 1.0 therefore provide evidence against differential surveillance, detection, or coding bias—a stronger validity check than negative-control outcomes that share pathways with the exposure or are surveillance-dependent.

Supplementary Figures

Figure S1. Graphical Representation of Study Design

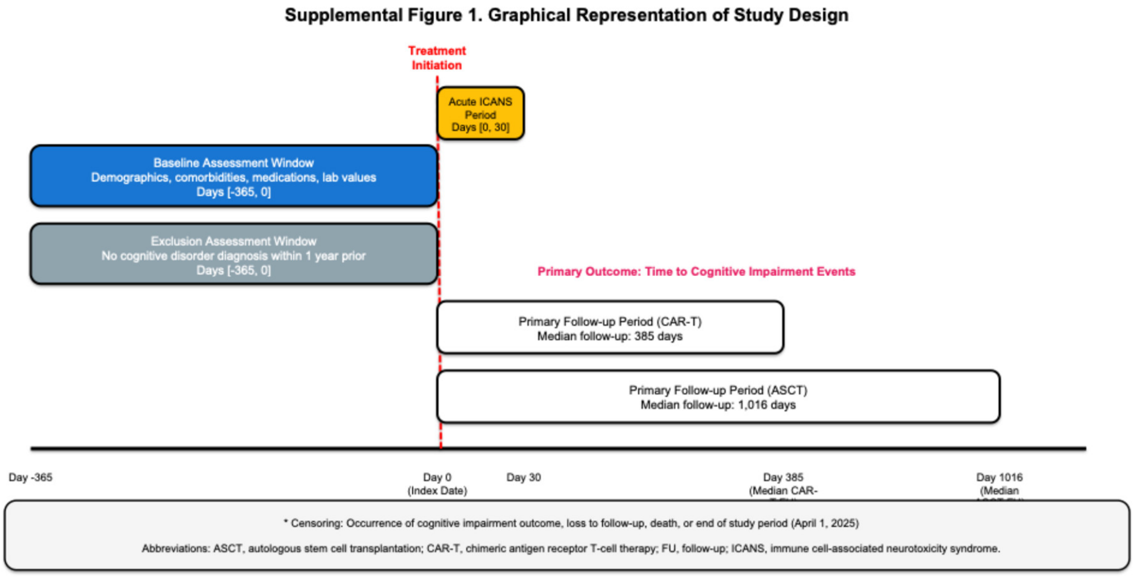

Figure S1 depicts the study timeline. The study period extended from 1 January 2014 through 1 April 2025. Patients were identified at the index date (CAR-T infusion or ASCT), with a 6-month pre-index baseline assessment window and follow-up until death, loss to follow-up (>180 days without an encounter), or end of study. Propensity score matching was performed at baseline to create balanced cohorts, and primary and secondary outcomes were assessed throughout follow-up.

Figure S2. Restricted Mean Survival Time (RMST) for Cognitive-Impairment–Free Survival

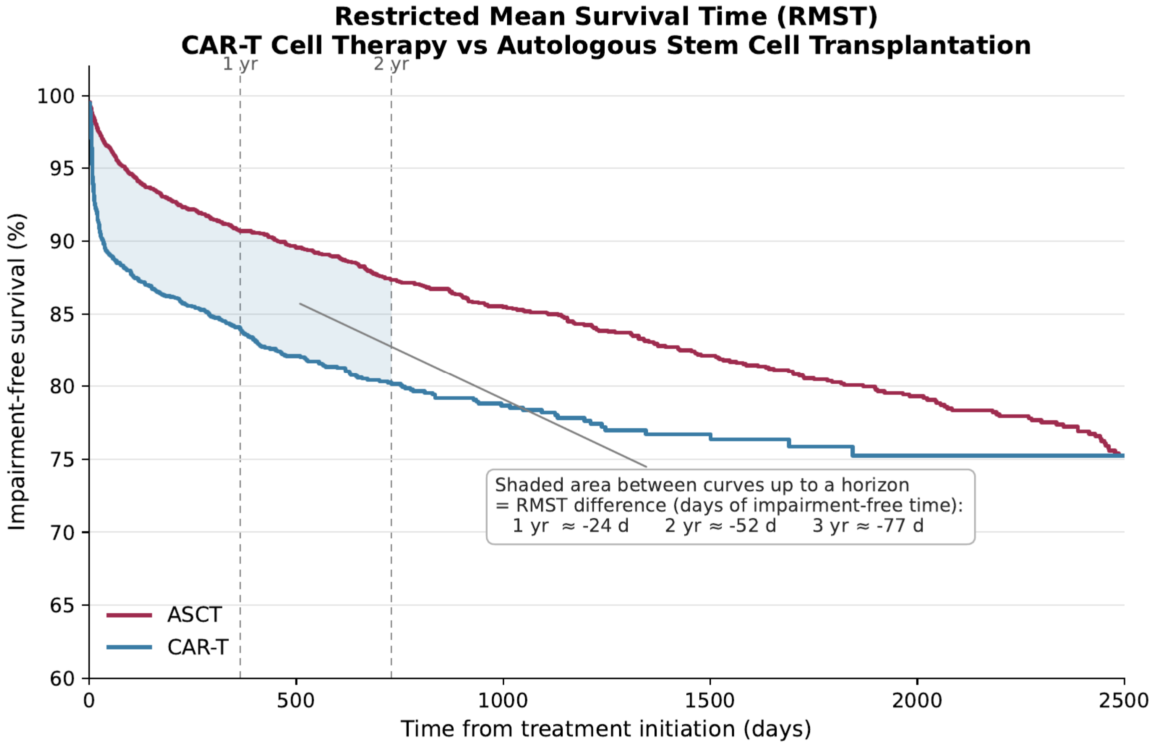

Figure S2. *Impairment-free survival (Kaplan–Meier) for CAR-T versus ASCT. The shaded area between the two curves up to a given horizon equals the restricted mean survival time (RMST) difference at that horizon (the difference in mean cognitive-impairment–free days). CAR-T recipients accrued fewer impairment-free days than ASCT recipients, with the difference widening over time (approximately −25 days at 1 year, −53 days at 2 years, and −80 days at 3 years; see Table 2c for point estimates with 95% confidence intervals). Because the proportional-hazards assumption was violated (Schoenfeld global test, P<0.001), RMST is presented as the principal, assumption-free effect measure.*

Supplementary Tables

Table S1. Complete ICD-10 Code Definitions for All Outcomes

ICD-10 Code	Description	Outcome Category
**Primary Outcome: Cognitive Impairment (composite)**		
R41.81	Age-related cognitive decline; mild cognitive impairment	Cognitive
R41.89	Other symptoms and signs involving cognitive functions and awareness	Cognitive
R41.3	Other amnesia; memory loss	Cognitive
R41.840	Attention and concentration deficit	Cognitive
R41.82	Altered mental status, unspecified	Cognitive
F03.90	Unspecified dementia without behavioral disturbance	Cognitive
R45.81	Apathy; low motivation	Cognitive
**Secondary Outcomes: Neurological Dysfunction**		
F05	Delirium due to known physiological condition	Neurological
G93.40–G93.49	Encephalopathy	Neurological
G40.0–G40.9	Epilepsy and recurrent seizures	Neurological
R56.9	Unspecified convulsions	Neurological
R51	Headache	Neurological
G43.0–G43.9	Migraine	Neurological
**Secondary Outcomes: Mood and Stress-Related Disorders**		
F32.0–F32.9	Depressive episode	Psychiatric
F33.0–F33.9	Major depressive disorder, recurrent	Psychiatric
F41.0–F41.9	Anxiety disorders	Psychiatric
G47.0–G47.9	Sleep disorders	Psychiatric
**Secondary Outcomes: Functional Impairment**		
R26.0–R26.9	Abnormalities of gait and mobility	Functional
W19, R29.6	Falls and repeated falls	Functional
**Secondary Outcomes: Hematologic Outcomes**		
C92.0–C92.9	Acute myeloid leukemia	Hematologic
D46.0–D46.9	Myelodysplastic syndrome	Hematologic
C84.0–C86.6	Mature T/NK-cell lymphoma (secondary T-cell lymphoma)	Hematologic
D65–D68.9	Coagulation defects (incl. DIC and other coagulation abnormality)	Hematologic
I26.0–I26.99, I80.0–I82.9	Pulmonary embolism and venous thrombosis/embolism (VTE)	Hematologic
**Negative Control Outcomes**		
K80.0–K80.2	Calculus of gallbladder	Negative Control
N20.0–N20.2	Calculus of kidney	Negative Control
K35.2–K35.8	Acute appendicitis	Negative Control

Note: *ICD-10-CM, International Classification of Diseases, Tenth Revision, Clinical Modification. Range notation (e.g., G93.40–G93.49) indicates that all subcodes within the category were included. The primary composite excludes acute confusional/delirium codes; delirium is reported separately as a neurological outcome. Code families for outcomes added during revision (delirium, secondary T-cell*

lymphoma, coagulation abnormality, venous thromboembolism) follow the corresponding ICD-10 chapters and the TriNetX outcome definitions.

Table S2. Sensitivity Analyses Excluding Cognitive-Impairment Events Within the Acute ICANS Window

Sensitivity analysis	HR (95% CI)	P value	E-value
Events within 7 days excluded	**1.695 (1.466–1.959)**	**<0.001**	2.78
Events within 14 days excluded	**1.428 (1.225–1.666)**	**<0.001**	2.21
Events within 21 days excluded	**1.330 (1.134–1.560)**	**<0.001**	1.99

Notes: *Each analysis excludes composite cognitive-impairment events occurring within the specified number of days after the index date and re-estimates the Cox hazard ratio over the remaining follow-up. The association remained significant across all windows. Immortal-time–corrected landmark analyses (Day 30 and Day 90), which re-anchor follow-up rather than simply excluding early events, are reported in main-text Table 2b. E-values denote the minimum strength of association an unmeasured confounder would require to explain the estimate.*

Abbreviations: *CI, confidence interval; HR, hazard ratio; ICANS, immune cell-associated neurotoxicity syndrome.*
